# Ethyl 1-benzyl-1,2,3,3a,4,10b-hexa­hydro­pyrrolo­[2′,3′:3,4]pyrrolo­[1,2-*a*]benzimidazole-2-carboxyl­ate

**DOI:** 10.1107/S1600536811014292

**Published:** 2011-04-29

**Authors:** Liping Meng, James C. Fettinger, Mark J. Kurth

**Affiliations:** a Department of Chemistry, University of California, One Shields Avenue, Davis, CA 95616, USA

## Abstract

The title mol­ecule, C_22_H_23_N_3_O_2_, was obtained *via* an intra­molecular cyclo­addition of an azomethine ylide and an alkene tethered by a benzimidazole unit. The benzoimidazole unit is essentially planar, with an r.m.s. deviation of 0.0087 Å from the nine constituent atoms. It has a *cis* fusion of the two pyrrolidine rings as well as a *cis* ester appendage. The two pyrrolidine rings rings have envelope conformations. The crystal packing is stabilized by aromatic π–π stacking of parallel benzimidazole ring systems, with a centroid-to-centroid distance of 3.518 (6) Å. Weak inter­molecular C—H⋯O contacts may also play a role in the stability of the packing.

## Related literature

Polycyclic nitro­gen-containing heterocycles form the basic skeleton of numerous alkaloids and physiologically active compounds, see: Southon & Buckingham (1989 ▶). Conformational studies have been reported for related pyrrolidino[3,4-*b*]pyrrolidine-2-carboxyl­ates obtained from intra­molecular cyclo­addition of azomethine ylides, see: Cheng *et al.* (2001 ▶); Meng *et al.* (2007 ▶). For related literature on the pharmaceutical properties of benzimidazole and pyrrolidine, see: Gudmundsson *et al.* (2000 ▶); Hamilton & Steiner (1997 ▶). For related literature on the azomethine ylide cycloaddition in similar systems, Pedrosa *et al.* (2006 ▶); Yang *et al.* (2006 ▶).
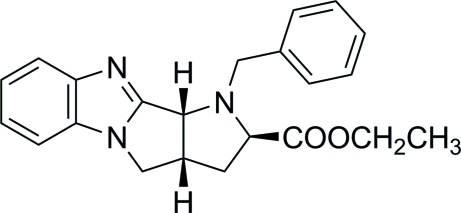


## Experimental

### 

#### Crystal data

C_22_H_23_N_3_O_2_
*M* *_r_* = 361.43
Monoclinic, 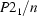

*a* = 9.2498 (5) Å
*b* = 13.8999 (7) Å
*c* = 14.2258 (7) Å
β = 90.345 (1)°
*V* = 1829.00 (16) Å^3^
*Z* = 4
Mo *K*α radiation
μ = 0.09 mm^−1^
*T* = 90 K
0.39 × 0.16 × 0.13 mm

#### Data collection

Bruker SMART1000 CCD area-detector diffractometer
Absorption correction: numerical (*SADABS*; Blessing, 1995 ▶; Sheldrick, 2007 ▶) *T*_min_ = 0.962, *T*_max_ = 0.989
16147 measured reflections
4194 independent reflections
3080 reflections with *I* > 2σ(*I*)
*R*_int_ = 0.037

#### Refinement

*R*[*F* ^2^ > 2σ(*F* ^2^)] = 0.037
*wR*(*F* ^2^) = 0.095
*S* = 1.09
4194 reflections
336 parameters
All H-atom parameters refined
Δρ_max_ = 0.37 e Å^−3^
Δρ_min_ = −0.27 e Å^−3^

### 

Data collection: *SMART* (Bruker, 2002 ▶); cell refinement: *SAINT* (Bruker, 2007 ▶); data reduction: *SAINT*; program(s) used to solve structure: *SHELXS97* (Sheldrick, 2008 ▶); program(s) used to refine structure: *SHELXL97* (Sheldrick, 2008 ▶); molecular graphics: *XP* in *SHELXTL/PC* (Sheldrick, 2008 ▶); software used to prepare material for publication: *SHELXL97*.

## Supplementary Material

Crystal structure: contains datablocks global, I. DOI: 10.1107/S1600536811014292/wn2427sup1.cif

Structure factors: contains datablocks I. DOI: 10.1107/S1600536811014292/wn2427Isup2.hkl

Additional supplementary materials:  crystallographic information; 3D view; checkCIF report

## Figures and Tables

**Table 1:** Hydrogen-bond geometry (Å, °)

*D*—H⋯*A*	*D*—H	H⋯*A*	*D*⋯*A*	*D*—H⋯*A*
C11—H11*B*⋯O12^i^	1.005 (15)	2.399 (15)	3.3344 (17)	154.5 (12)
C18—H18⋯O12^ii^	0.968 (15)	2.514 (16)	3.3505 (17)	144.6 (12)

Symmetry codes: (i) 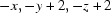; (ii) 
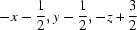
.

**Fig. 1.**
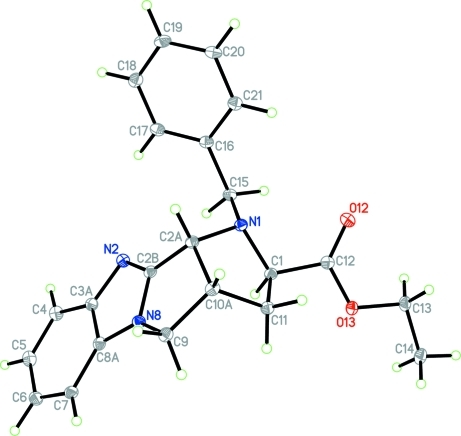
The molecular structure of the title compound. Ellipsoids are drawn at the 50% probability level. H atoms are represented as small spheres of arbitrary radius.

**Fig. 2.**
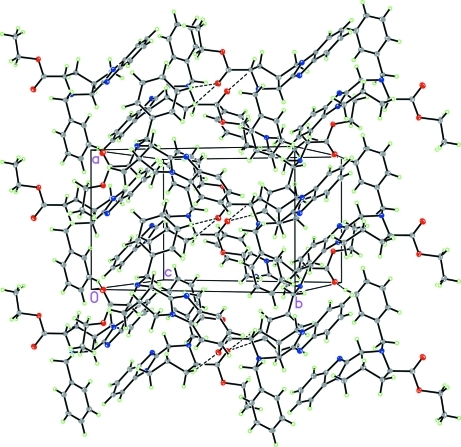
The crystal packing of the title compound with a view of the C—H···O (dashed lines) and π–π interactions.

## supplementary crystallographic information

### Comment

Polycyclic nitrogen-containing heterocycles form the basic skeleton of numerous alkaloids and physiologically active compounds (Southon & Buckingham, 1989). The title polycyclic *N*-heterocycle, ethyl 1-benzylpyrrolidino[2',3':3,4]pyrrolidino[1,2-*a*] benzimidazole-2-carboxylate, was obtained *via* an intramolecular azomethine ylide cycloaddition and possesses two medicinally relevant pharmacophores – benzimidazole and pyrrolidine (Gudmundsson *et al.*, 2000; Hamilton & Steiner, 1997)– in one rigid molecule; the title compound may afford important bioactivity.

In the structure of the title compound (Fig. 1), the benzoimidazole unit is essentially planar, with a root mean square deviation of 0.0087 Å from the nine constituent atoms. The two pyrrolidine rings have envelope forms and are *cis* fused, which is consistent with conventional azomethine ylide cycloadditions in similar systems (Pedrosa *et al.*, 2006; Yang *et al.*, 2006). However, unlike the previously reported analogues obtained from an intramolecular azomethine ylide and alkene cycloaddition tethered by an oxazolidin-2-one (Cheng *et al.*, 2001), the ester appendage in the title structure was unambiguously assigned as *cis* to the angular protons H2A and H10A by X-ray crystallography. The crystal packing (Fig. 2) exhibits π–π stacking of parallel benzimidazole ring systems, with a *Cg*1···*Cg*2 distance of 3.518 Å [*Cg*1 and *Cg*2 are the centroids of the C3A–C8A benzene ring in one molecule and the C2B/N2/C3A/C8A/N8 imidazole ring in the other molecule, respectively]. Intermolecular C—H···O contacts may also play a role in the stability of the packing.

### Experimental

The title compound was prepared from 1-allyl-1*H*-benzimidazole-2-carbaldehyde, *N*-benzylglycine ethyl ester hydrochloride, and triethylamine according to the procedure reported by Meng *et al.* (2007). Colourless blocks of the title compound were obtained by recrystallization from EtOAc/n-hexane 1:1 with slow evaporation at room temperature. These crystals were suitable for X-ray crystallography.

### Refinement

H atoms were located directly in a difference Fourier map and then allowed to refine freely throughout the final convergence stage. The final structure was refined to convergence [Δ/σ≤ 0.001]. The final difference Fourier map was featureless, indicating that the structure is both correct and complete.

### Crystal data

**Table:**

C_22_H_23_N_3_O_2_	*F*(000) = 768
*M**_r_* = 361.43	*D*_x_ = 1.313 Mg m^−^^3^
Monoclinic, *P*2_1_/*n*	Mo *K*α radiation, λ = 0.71073 Å
Hall symbol: -P 2yn	Cell parameters from 7133 reflections
*a* = 9.2498 (5) Å	θ = 2.6–27.5°
*b* = 13.8999 (7) Å	µ = 0.09 mm^−^^1^
*c* = 14.2258 (7) Å	*T* = 90 K
β = 90.345 (1)°	Block, colourless
*V* = 1829.00 (16) Å^3^	0.39 × 0.16 × 0.13 mm
*Z* = 4	

### Data collection

**Table:**

Bruker SMART1000 CCD area-detector diffractometer	4194 independent reflections
Radiation source: fine-focus sealed tube	3080 reflections with *I* > 2σ(*I*)
graphite	*R*_int_ = 0.037
ω and φ scans	θ_max_ = 27.5°, θ_min_ = 2.1°
Absorption correction: numerical (*SADABS*; Blessing, 1995; Sheldrick, 2007)	*h* = −12→12
*T*_min_ = 0.962, *T*_max_ = 0.989	*k* = −18→18
16147 measured reflections	*l* = −18→18

### Refinement

**Table:**

Refinement on *F*^2^	Primary atom site location: structure-invariant direct methods
Least-squares matrix: full	Secondary atom site location: difference Fourier map
*R*[*F*^2^ > 2σ(*F*^2^)] = 0.037	Hydrogen site location: inferred from neighbouring sites
*wR*(*F*^2^) = 0.095	All H-atom parameters refined
*S* = 1.09	*w* = 1/[σ^2^(*F*_o_^2^) + (0.0417*P*)^2^ + 0.3839*P*] where *P* = (*F*_o_^2^ + 2*F*_c_^2^)/3
4194 reflections	(Δ/σ)_max_ = 0.001
336 parameters	Δρ_max_ = 0.37 e Å^−^^3^
0 restraints	Δρ_min_ = −0.27 e Å^−^^3^

### Special details

**Table:**

Experimental. A colourless block with approximate orthogonal dimensions 0.39 × 0.16 × 0.13 mm^3^ was placed and optically centered on the Bruker SMART1000 CCD system at -183°C. The initial unit cell was indexed using a least-squares analysis of a random set of reflections collected from three series of 0.3° wide ω scans, 10 s per frame, and 25 frames per series that were well distributed in reciprocal space. Four ω-scan data frame series were collected [Mo *K*α] with 0.3° wide scans, 30 s per frame and 606, 435, 606, 435 frames collected per series at varying φ angles (φ = 0°, 90°, 180°, 270°), respectively.
Geometry. All e.s.d.'s (except the e.s.d. in the dihedral angle between two l.s. planes) are estimated using the full covariance matrix. The cell e.s.d.'s are taken into account individually in the estimation of e.s.d.'s in distances, angles and torsion angles; correlations between e.s.d.'s in cell parameters are only used when they are defined by crystal symmetry. An approximate (isotropic) treatment of cell e.s.d.'s is used for estimating e.s.d.'s involving l.s. planes.
Refinement. The crystal to detector distance was 4.123 cm, thus providing a complete sphere of data to 2θ_max_ = 55.06°. A total of 23465 reflections were collected and corrected for Lorentz and polarization effects and absorption using Blessing's method (Blessing, 1995) as incorporated into the program *SADABS* with 4390 unique. All crystallographic calculations were performed on a personal computer (PC) with a Pentium 3.20 GHz processor and 1 GB of extended memory. The *SHELXTL* program package was implemented to determine the probable space group and set up the initial files. System symmetry, systematic absences and intensity statistics indicated the centrosymmetric monoclinic non-standard space group *P*2_1_/*n* (No. 14). The structure was determined by direct methods with the successful location of a majority of the molecule within the asymmetric unit using the program XS. The structure was refined with XL. The 23465 data collected were merged based upon identical indices yielding 16536 data [*R*(int) = 0.0245] that were truncated to 2θ_max_ = 55.0° resulting in 16147 data that were further merged during least-squares refinement to 4194 unique data [*R*(int) = 0.0373]. A single least-squares difference Fourier cycle was required to locate the remaining non-H atoms. All non-H atoms were refined anisotropically. Refinement of *F*^2^ against ALL reflections. The weighted *R*-factor *wR* and goodness of fit *S* are based on *F*^2^, conventional *R*-factors *R* are based on *F*, with *F* set to zero for negative *F*^2^. The threshold expression of *F*^2^ > σ(*F*^2^) is used only for calculating *R*-factors(gt) *etc*. and is not relevant to the choice of reflections for refinement. *R*-factors based on *F*^2^ are statistically about twice as large as those based on *F*, and *R*-factors based on ALL data will be even larger.

### Fractional atomic coordinates and isotropic or equivalent isotropic displacement parameters (Å^2^)

**Table:**

	*x*	*y*	*z*	*U*_iso_*/*U*_eq_	
C1	0.11370 (13)	0.83406 (9)	0.93388 (9)	0.0171 (3)	
H1	0.1841 (15)	0.8014 (10)	0.8905 (10)	0.016 (3)*	
N1	−0.03076 (11)	0.79245 (7)	0.92753 (7)	0.0167 (2)	
C2A	−0.02373 (14)	0.70281 (9)	0.98214 (9)	0.0168 (3)	
H2A	−0.1230 (16)	0.6828 (10)	0.9972 (10)	0.021 (4)*	
C2B	0.05845 (13)	0.61970 (9)	0.93939 (9)	0.0164 (3)	
N2	0.04725 (11)	0.56851 (8)	0.86159 (7)	0.0188 (2)	
C3A	0.15526 (14)	0.49885 (9)	0.87233 (9)	0.0178 (3)	
C4	0.19276 (15)	0.42310 (10)	0.81285 (9)	0.0215 (3)	
H4	0.1418 (16)	0.4118 (10)	0.7547 (11)	0.024 (4)*	
C5	0.30288 (15)	0.36208 (10)	0.84115 (10)	0.0239 (3)	
H5	0.3296 (16)	0.3071 (11)	0.8029 (11)	0.024 (4)*	
C6	0.37646 (15)	0.37548 (10)	0.92687 (10)	0.0231 (3)	
H6	0.4508 (16)	0.3301 (11)	0.9460 (10)	0.025 (4)*	
C7	0.34082 (14)	0.44980 (10)	0.98730 (10)	0.0205 (3)	
H7	0.3892 (16)	0.4588 (10)	1.0466 (11)	0.023 (4)*	
C8A	0.22957 (14)	0.51062 (9)	0.95859 (9)	0.0176 (3)	
N8	0.16544 (11)	0.58997 (8)	0.99883 (7)	0.0173 (2)	
C9	0.16669 (15)	0.63878 (10)	1.08991 (9)	0.0191 (3)	
H9A	0.2688 (16)	0.6555 (10)	1.1086 (10)	0.021 (4)*	
H9B	0.1242 (15)	0.5967 (10)	1.1379 (10)	0.022 (4)*	
C10A	0.07245 (14)	0.72792 (9)	1.06910 (9)	0.0180 (3)	
H10A	0.0137 (14)	0.7435 (9)	1.1239 (10)	0.013 (3)*	
C11	0.15928 (15)	0.81681 (10)	1.03687 (9)	0.0193 (3)	
H11A	0.2653 (18)	0.8059 (11)	1.0440 (11)	0.029 (4)*	
H11B	0.1335 (16)	0.8755 (11)	1.0745 (11)	0.025 (4)*	
C12	0.11502 (14)	0.93996 (9)	0.90881 (9)	0.0182 (3)	
O12	0.01249 (10)	0.98789 (7)	0.88602 (7)	0.0248 (2)	
C13	0.27147 (16)	1.07485 (10)	0.89143 (10)	0.0225 (3)	
H13A	0.2190 (16)	1.1145 (11)	0.9382 (11)	0.023 (4)*	
H13B	0.2268 (16)	1.0867 (10)	0.8302 (11)	0.022 (4)*	
O13	0.25101 (10)	0.97384 (6)	0.91573 (6)	0.0201 (2)	
C14	0.43191 (16)	1.09273 (11)	0.89238 (11)	0.0251 (3)	
H14A	0.4769 (18)	1.0798 (12)	0.9539 (13)	0.037 (5)*	
H14B	0.4516 (18)	1.1615 (13)	0.8784 (11)	0.038 (5)*	
H14C	0.4819 (17)	1.0527 (12)	0.8441 (12)	0.035 (4)*	
C15	−0.08571 (14)	0.78122 (10)	0.83087 (9)	0.0179 (3)	
H15A	−0.0297 (15)	0.7327 (10)	0.7948 (10)	0.018 (4)*	
H15B	−0.0739 (15)	0.8460 (11)	0.8007 (10)	0.018 (4)*	
C16	−0.24288 (14)	0.75165 (9)	0.82970 (9)	0.0178 (3)	
C17	−0.28700 (15)	0.66465 (10)	0.79108 (9)	0.0205 (3)	
H17	−0.2161 (17)	0.6209 (11)	0.7651 (11)	0.030 (4)*	
C18	−0.43276 (15)	0.63824 (10)	0.79093 (9)	0.0228 (3)	
H18	−0.4629 (16)	0.5773 (11)	0.7644 (11)	0.027 (4)*	
C19	−0.53434 (15)	0.69891 (11)	0.83067 (9)	0.0232 (3)	
H19	−0.6361 (17)	0.6801 (11)	0.8303 (10)	0.024 (4)*	
C20	−0.49157 (15)	0.78651 (10)	0.86906 (9)	0.0224 (3)	
H20	−0.5630 (16)	0.8300 (11)	0.8959 (10)	0.022 (4)*	
C21	−0.34683 (14)	0.81280 (10)	0.86831 (9)	0.0205 (3)	
H21	−0.3146 (16)	0.8746 (11)	0.8952 (10)	0.022 (4)*	

### Atomic displacement parameters (Å^2^)

**Table:**

	*U*^11^	*U*^22^	*U*^33^	*U*^12^	*U*^13^	*U*^23^
C1	0.0135 (6)	0.0187 (6)	0.0189 (6)	0.0002 (5)	0.0018 (5)	−0.0014 (5)
N1	0.0149 (5)	0.0182 (6)	0.0170 (5)	−0.0009 (4)	−0.0002 (4)	0.0011 (4)
C2A	0.0146 (6)	0.0196 (7)	0.0161 (6)	−0.0012 (5)	0.0012 (5)	0.0005 (5)
C2B	0.0145 (6)	0.0186 (6)	0.0162 (6)	−0.0027 (5)	0.0009 (5)	0.0025 (5)
N2	0.0194 (6)	0.0191 (6)	0.0180 (5)	0.0006 (4)	0.0011 (4)	0.0000 (4)
C3A	0.0165 (6)	0.0193 (6)	0.0177 (6)	−0.0013 (5)	0.0030 (5)	0.0042 (5)
C4	0.0244 (7)	0.0236 (7)	0.0164 (7)	−0.0002 (6)	0.0044 (5)	0.0006 (5)
C5	0.0251 (7)	0.0226 (7)	0.0242 (7)	0.0017 (6)	0.0096 (6)	0.0001 (6)
C6	0.0172 (7)	0.0221 (7)	0.0300 (8)	0.0013 (6)	0.0049 (6)	0.0053 (6)
C7	0.0159 (7)	0.0225 (7)	0.0230 (7)	−0.0026 (5)	0.0004 (5)	0.0050 (5)
C8A	0.0173 (6)	0.0179 (6)	0.0177 (6)	−0.0027 (5)	0.0028 (5)	0.0026 (5)
N8	0.0170 (6)	0.0184 (5)	0.0165 (5)	−0.0004 (4)	−0.0005 (4)	0.0013 (4)
C9	0.0207 (7)	0.0208 (7)	0.0157 (6)	−0.0023 (5)	−0.0014 (5)	−0.0002 (5)
C10A	0.0179 (6)	0.0216 (7)	0.0146 (6)	−0.0013 (5)	0.0022 (5)	−0.0014 (5)
C11	0.0194 (7)	0.0182 (7)	0.0204 (7)	−0.0005 (5)	−0.0029 (5)	0.0004 (5)
C12	0.0187 (7)	0.0213 (7)	0.0146 (6)	0.0013 (5)	0.0022 (5)	−0.0021 (5)
O12	0.0232 (5)	0.0226 (5)	0.0284 (5)	0.0044 (4)	−0.0022 (4)	0.0015 (4)
C13	0.0289 (8)	0.0154 (7)	0.0233 (7)	−0.0024 (6)	−0.0026 (6)	0.0023 (5)
O13	0.0207 (5)	0.0161 (5)	0.0235 (5)	−0.0020 (4)	0.0003 (4)	0.0015 (4)
C14	0.0297 (8)	0.0199 (7)	0.0257 (8)	−0.0066 (6)	−0.0002 (6)	0.0008 (6)
C15	0.0159 (6)	0.0221 (7)	0.0156 (6)	0.0008 (5)	0.0010 (5)	0.0000 (5)
C16	0.0173 (7)	0.0229 (7)	0.0130 (6)	0.0012 (5)	−0.0013 (5)	0.0031 (5)
C17	0.0201 (7)	0.0248 (7)	0.0164 (6)	0.0032 (6)	−0.0009 (5)	0.0009 (5)
C18	0.0242 (7)	0.0247 (7)	0.0193 (7)	−0.0027 (6)	−0.0052 (5)	0.0014 (6)
C19	0.0165 (7)	0.0322 (8)	0.0209 (7)	−0.0019 (6)	−0.0033 (5)	0.0043 (6)
C20	0.0171 (7)	0.0305 (8)	0.0195 (7)	0.0047 (6)	−0.0005 (5)	0.0010 (6)
C21	0.0206 (7)	0.0232 (7)	0.0177 (6)	0.0010 (6)	−0.0011 (5)	−0.0008 (5)

### Geometric parameters (Å, °)

**Table:**

C1—N1	1.4582 (16)	C10A—C11	1.5448 (18)
C1—C12	1.5147 (18)	C10A—H10A	0.977 (14)
C1—C11	1.5409 (18)	C11—H11A	0.997 (16)
C1—H1	1.008 (14)	C11—H11B	1.005 (15)
N1—C2A	1.4696 (16)	C12—O12	1.2019 (16)
N1—C15	1.4714 (16)	C12—O13	1.3462 (15)
C2A—C2B	1.5126 (18)	C13—O13	1.4584 (16)
C2A—C10A	1.5590 (17)	C13—C14	1.505 (2)
C2A—H2A	0.984 (15)	C13—H13A	0.993 (15)
C2B—N2	1.3193 (16)	C13—H13B	0.976 (15)
C2B—N8	1.3620 (16)	C14—H14A	0.983 (18)
N2—C3A	1.3990 (17)	C14—H14B	0.994 (18)
C3A—C4	1.3959 (18)	C14—H14C	0.999 (17)
C3A—C8A	1.4125 (18)	C15—C16	1.5108 (18)
C4—C5	1.383 (2)	C15—H15A	0.994 (14)
C4—H4	0.963 (16)	C15—H15B	1.004 (15)
C5—C6	1.405 (2)	C16—C17	1.3885 (19)
C5—H5	0.971 (15)	C16—C21	1.3982 (18)
C6—C7	1.385 (2)	C17—C18	1.3973 (19)
C6—H6	0.971 (16)	C17—H17	0.969 (16)
C7—C8A	1.3912 (18)	C18—C19	1.386 (2)
C7—H7	0.961 (15)	C18—H18	0.967 (16)
C8A—N8	1.3785 (16)	C19—C20	1.391 (2)
N8—C9	1.4625 (16)	C19—H19	0.977 (15)
C9—C10A	1.5425 (18)	C20—C21	1.3879 (19)
C9—H9A	1.006 (15)	C20—H20	0.975 (15)
C9—H9B	0.983 (15)	C21—H21	0.986 (15)
			
N1—C1—C12	112.30 (10)	C9—C10A—H10A	110.0 (8)
N1—C1—C11	104.04 (10)	C11—C10A—H10A	110.6 (8)
C12—C1—C11	111.87 (11)	C2A—C10A—H10A	111.4 (8)
N1—C1—H1	112.2 (8)	C1—C11—C10A	105.48 (10)
C12—C1—H1	106.7 (8)	C1—C11—H11A	112.6 (9)
C11—C1—H1	109.7 (8)	C10A—C11—H11A	111.2 (9)
C1—N1—C2A	105.42 (10)	C1—C11—H11B	108.4 (9)
C1—N1—C15	114.29 (10)	C10A—C11—H11B	111.5 (9)
C2A—N1—C15	114.70 (10)	H11A—C11—H11B	107.8 (12)
N1—C2A—C2B	117.12 (10)	O12—C12—O13	124.14 (12)
N1—C2A—C10A	104.65 (10)	O12—C12—C1	126.49 (12)
C2B—C2A—C10A	101.79 (10)	O13—C12—C1	109.37 (11)
N1—C2A—H2A	108.5 (8)	O13—C13—C14	106.64 (11)
C2B—C2A—H2A	110.1 (9)	O13—C13—H13A	108.1 (9)
C10A—C2A—H2A	114.8 (8)	C14—C13—H13A	112.9 (9)
N2—C2B—N8	114.23 (11)	O13—C13—H13B	108.6 (9)
N2—C2B—C2A	135.40 (12)	C14—C13—H13B	113.1 (9)
N8—C2B—C2A	110.31 (11)	H13A—C13—H13B	107.4 (12)
C2B—N2—C3A	103.25 (11)	C12—O13—C13	116.23 (10)
C4—C3A—N2	129.48 (12)	C13—C14—H14A	112.9 (10)
C4—C3A—C8A	119.52 (12)	C13—C14—H14B	109.8 (10)
N2—C3A—C8A	110.99 (11)	H14A—C14—H14B	106.1 (14)
C5—C4—C3A	118.11 (13)	C13—C14—H14C	111.3 (10)
C5—C4—H4	120.4 (9)	H14A—C14—H14C	108.3 (14)
C3A—C4—H4	121.5 (9)	H14B—C14—H14C	108.2 (13)
C4—C5—C6	121.55 (13)	N1—C15—C16	111.48 (10)
C4—C5—H5	120.6 (9)	N1—C15—H15A	112.1 (8)
C6—C5—H5	117.8 (9)	C16—C15—H15A	108.3 (8)
C7—C6—C5	121.44 (13)	N1—C15—H15B	105.5 (8)
C7—C6—H6	118.8 (9)	C16—C15—H15B	110.3 (8)
C5—C6—H6	119.7 (9)	H15A—C15—H15B	109.2 (11)
C6—C7—C8A	116.70 (13)	C17—C16—C21	118.93 (12)
C6—C7—H7	122.1 (9)	C17—C16—C15	121.45 (12)
C8A—C7—H7	121.2 (9)	C21—C16—C15	119.62 (12)
N8—C8A—C7	133.13 (12)	C16—C17—C18	120.71 (13)
N8—C8A—C3A	104.19 (11)	C16—C17—H17	119.9 (9)
C7—C8A—C3A	122.68 (12)	C18—C17—H17	119.3 (9)
C2B—N8—C8A	107.32 (10)	C19—C18—C17	119.74 (13)
C2B—N8—C9	114.28 (11)	C19—C18—H18	119.8 (9)
C8A—N8—C9	137.56 (11)	C17—C18—H18	120.4 (9)
N8—C9—C10A	101.61 (10)	C18—C19—C20	120.07 (13)
N8—C9—H9A	110.0 (8)	C18—C19—H19	119.4 (9)
C10A—C9—H9A	113.2 (8)	C20—C19—H19	120.6 (9)
N8—C9—H9B	109.8 (9)	C21—C20—C19	119.96 (13)
C10A—C9—H9B	112.6 (9)	C21—C20—H20	119.7 (9)
H9A—C9—H9B	109.4 (12)	C19—C20—H20	120.3 (9)
C9—C10A—C11	113.90 (11)	C20—C21—C16	120.58 (13)
C9—C10A—C2A	106.95 (10)	C20—C21—H21	121.1 (9)
C11—C10A—C2A	103.81 (10)	C16—C21—H21	118.4 (9)

### Hydrogen-bond geometry (Å, °)

**Table:**

*D*—H···*A*	*D*—H	H···*A*	*D*···*A*	*D*—H···*A*
C11—H11B···O12^i^	1.005 (15)	2.399 (15)	3.3344 (17)	154.5 (12)
C18—H18···O12^ii^	0.968 (15)	2.514 (16)	3.3505 (17)	144.6 (12)

Symmetry codes: (i) −*x*, −*y*+2, −*z*+2; (ii) −*x*−1/2, *y*−1/2, −*z*+3/2.
